# Spectrum of gynecologic malignancies in Northeastern Nigeria

**DOI:** 10.3389/fonc.2025.1420113

**Published:** 2025-03-18

**Authors:** Dauda A. Katagum, Uchenna S. Ezenkwa, Sunday E. Achanya, Aliyu I. Lawan, Dauda E. Suleiman, Mairo U. Kadaura, Abba Kabir, Adamu I. Adamu, Abubakar Kolomi Modu, Hadiza Usman, Sophia H. L. George, Matthew Schlumbrecht, Bala M. Audu

**Affiliations:** ^1^ Department of Obstetrics and Gynecology, Federal University of Health Sciences/Federal Medical Center, Azare, Nigeria; ^2^ Department of Histopathology, Federal University of Health Sciences/Federal Medical Center Azare, Azare, Nigeria; ^3^ Department of Histopathology, College of Medical Sciences, Gombe State University/Federal Teaching Hospital, Gombe, Nigeria; ^4^ Department of Histopathology, College of Medical Sciences, Abubakar Tafawa Balewa University, Bauchi, Nigeria; ^5^ Department of Clinical Microbiology, Federal University of Health Sciences, Azare, Nigeria; ^6^ Department of Histopathology, College of Medical Sciences, University of Maiduguri/University of Maiduguri Teaching Hospital, Maiduguri, Nigeria; ^7^ Department of Histopathology, Yobe State University/Yobe State University Teaching Hospital, Damaturu, Nigeria; ^8^ Department of Pathology, Federal Medical Center Nguru, Nguru, Nigeria; ^9^ Department of Obstetrics and Gynecology, Federal Medical Center Nguru, Nguru, Nigeria; ^10^ Obstetrics, Gynecology and Reproductive Sciences, Division of Gynecologic Oncology, Sylvester Comprehensive Cancer Center, University of Miami Miller School of Medicine, Miami, FL, United States

**Keywords:** gynecologic malignancy, cervical cancer, uterine cancer, ovary cancer, female genital cancers, Northeastern Nigeria

## Abstract

**Background:**

The burden of female genital tract cancers in low—and middle-income countries (LIMC) is not yet well investigated. Although available studies are few, they are mainly based on single institutions. Here, four-year multi-institutional data on gynecologic cancers in northeastern Nigeria were examined to determine their distribution by age and subtype.

**Patient and Methods:**

This is a cross-sectional descriptive study using available data on histologically diagnosed gynecologic cancers archived in the pathology departments and/or cancer registries of six tertiary hospitals in northeastern Nigeria over four years. Alongside tumor type (according to site), patient age and presenting complaints were also documented. Descriptive statistics were used to present categorical variables as proportions, while quantitative variables of age were presented as medians.

**Results:**

A total of 863 gynecologic cancers were included in this study. The median age was 50 years (3 – 95 years) with a peak at 40 – 49 years. The uterine cervix was the most common site of cancer (66.6%, 575/863), while uterine corpus (15.5%, 134/863) and ovarian cancers (14.8%; 128/863) were nearly equal in proportion. Squamous cell cancer was the commonest histotype overall, while ovarian cancers had a preponderance of epithelial tumors in 67.9% (87/128) over sex cord stromal (12.5%; 16/128) and germ cell (9.4%, 12/128) tumors. The most common symptom was abnormal vaginal bleeding (38%) followed by abdominal swelling (21.1%) and foul-smelling vaginal discharge (14.1%). Population-based data also showed high parity among women in the region, ranging from 4 births to 7 births per woman in a reproductive lifetime (total fertility rate, TFR).

**Conclusion:**

The spectrum of cancers of the female genital tract in this study mirrors the population demographics characterized by a high proportion of young women in their reproductive age. Efforts to reduce the burden of this disease are urgently warranted.

## Introduction

Gynecological cancers (GC) are a significant public health concern globally. They affect the female reproductive system such as the vulva, vagina, cervix, uterus, fallopian tubes and ovaries. Recent global estimates report that about 1,473,427 new cases of GCs and 680,372 deaths occur annually (1). Risk factors vary depending on the type of cancer, and despite advancements in prevention, diagnosis and treatment, metastasis and recurrence remain a significant challenge in the disease control effort. Hence, early detection through screening and awareness of symptoms is crucial for improved outcomes (2).

Africa has the highest burden of GC, specifically, East Africa, with an age-standardized incidence rate (ASIR) of 50 per 100,000 person-years compared to the global rate of 30.3 per 100,000 person-years (1). Examining the component cancers reveals differences in predisposing factors. For example, cervical, vaginal and vulva cancers share in common an interplay between Human papillomavirus (HPV) infection and modifying lifestyle behaviors such as early age at sexual debut, multiple sexual partners, and cigarette smoking (3, 4). Multiparity, dietary habits and exposure to talcum powder are factors significantly associated with ovarian cancer, while hormonal influence and genetic predispositions contribute to endometrial cancer (5–7). These varying risk factors highlight the need for appropriate screening and diagnostic interventions at proper ages to prevent or treat the disease. Unfortunately, low- and middle-income countries (LMICs) continue to suffer from these cancers due to inadequate screening and treatment services in place to manage them.

GC affects women in diverse ways, either from the disease or its treatment. Changes in body image, sexual identity and perceived desirability, for instance, can strain social relations and ties (8). Also, fertility and reproductive functioning can be compromised, raising worries for the patient (9). These, in addition to the huge financial burden of care, can induce anxiety and depression in the patient (10)

To drive the needed policy towards reducing or eradicating the burden of GC in LMICs, credible epidemiological data reporting is pivotal. Nigeria, like other sub-Saharan African countries, is still grappling with a high burden of communicable diseases (11). However, accumulating evidence shows that incidence of NCDs are on the rise, even though poorly documented (12). Studies describing comprehensive aggregate national data on GC in the country are rare. Available data are single-institution based, often describing a single GC disease component (13–15). This underscores the need for a study to collectively document these diseases on a regional and nationwide level for effective policy formulation and interventional strategies.

The present study used multi-institutional data on GC to determine the disease burden in northeastern Nigeria. It highlighted existing gaps and proposed remedies to address care disparities in the region. The findings are hoped to be relevant to further discussions on GC care approaches in the region and similar populations elsewhere.

## Patients and methods

A review of available histopathological data on gynecological cancers diagnosed over 4 years (January 2019 – December 2022) in tertiary hospitals within northeastern Nigeria was conducted retrospectively. The zone has six states – Adamawa, Bauchi, Borno, Gombe, Taraba and Yobe – with eight tertiary hospitals, Bauchi and Yobe having two each. Data retrieved included cancer organ or site, (uterine cervix, uterine corpus, fallopian tube, ovary, vagina, and vulva), patient age at diagnosis, and presenting complaints. All cases diagnosed within the study period were eligible for inclusion in the study, while all cases outside this range were excluded. Accrued data were reviewed by the pathologists in each of the contributing centers for conformity and reliability of information. When indicated, the histology slides were retrieved and examined using a light microscope to ascertain the proper classification of the cancer. Cases with doubtful classification and for which the slides were not available for review were further excluded. However, cases obtained from cancer registries were checked for accuracy of documentation as” cancer” and to ensure that they arose from the female reproductive tract. Otherwise, they were excluded.

The female population demographics for the geographical area were derived from the National Population Commission statistics report for 2020 (16). All required ethical and institutional approvals were obtained from the participating institutions. At the same time, the tumors were histologically classified based on the 5^th^ Edition of the World Health Organization protocol on the classification of tumors of the female genital tracts (17). Fallopian tube cancers were classified together with ovarian cancers, as recommended by the International Federation of Gynecology and Obstetrics (FIGO) (18).

Patient data was deidentified before retrieval and analysis. Frequency statistics was used to categorize nominal and ordinal variables into proportions and percentages while the median age of patients was calculated using measures of central tendency (median) statistical tool. The results are presented as tables, figures and in textual formats.

## Results

### Population of interest demographics in the Northeast zone

Six tertiary hospitals from four states in the region provided data for this study (**Supplementary 1**). These hospitals include the University of Maiduguri Teaching Hospital Borno State, Yobe State University Teaching Hospital, Yobe State, Federal Medical Center Nguru, Yobe State, Federal Teaching Hospital Gombe State, Federal Medical Center Azare, Bauchi State, and Abubakar Tafawa Balewa University Teaching Hospital, Bauchi State. Together, they serve a population of about 22 million people, females making up about 49.4%. The population distribution of the states from which data were drawn is shown in **Supplementary 2**. Two landmark age groups are displayed: 0 -49 years representing birth, early childhood and reproductive age groups; while 50 - 80+ years encompasses the period of fertility decline, menopause, and older age characterized by hormone replacement use, and upsurge in the risk for those malignancies that are dependent on hormone and genetics as risk factors. Illustrated in **Supplementary 3** is the Total Fertility Rate (TFR) for the women throughout their expected fertility years and describes a decline from the year 2008 through to the period under review (2019 – 2022). Borno state had the most decline followed by Yobe state while Bauchi state had the least reduction in fertility rate.

### Cancer data by sites, age and histological variants

**Figure 1:**
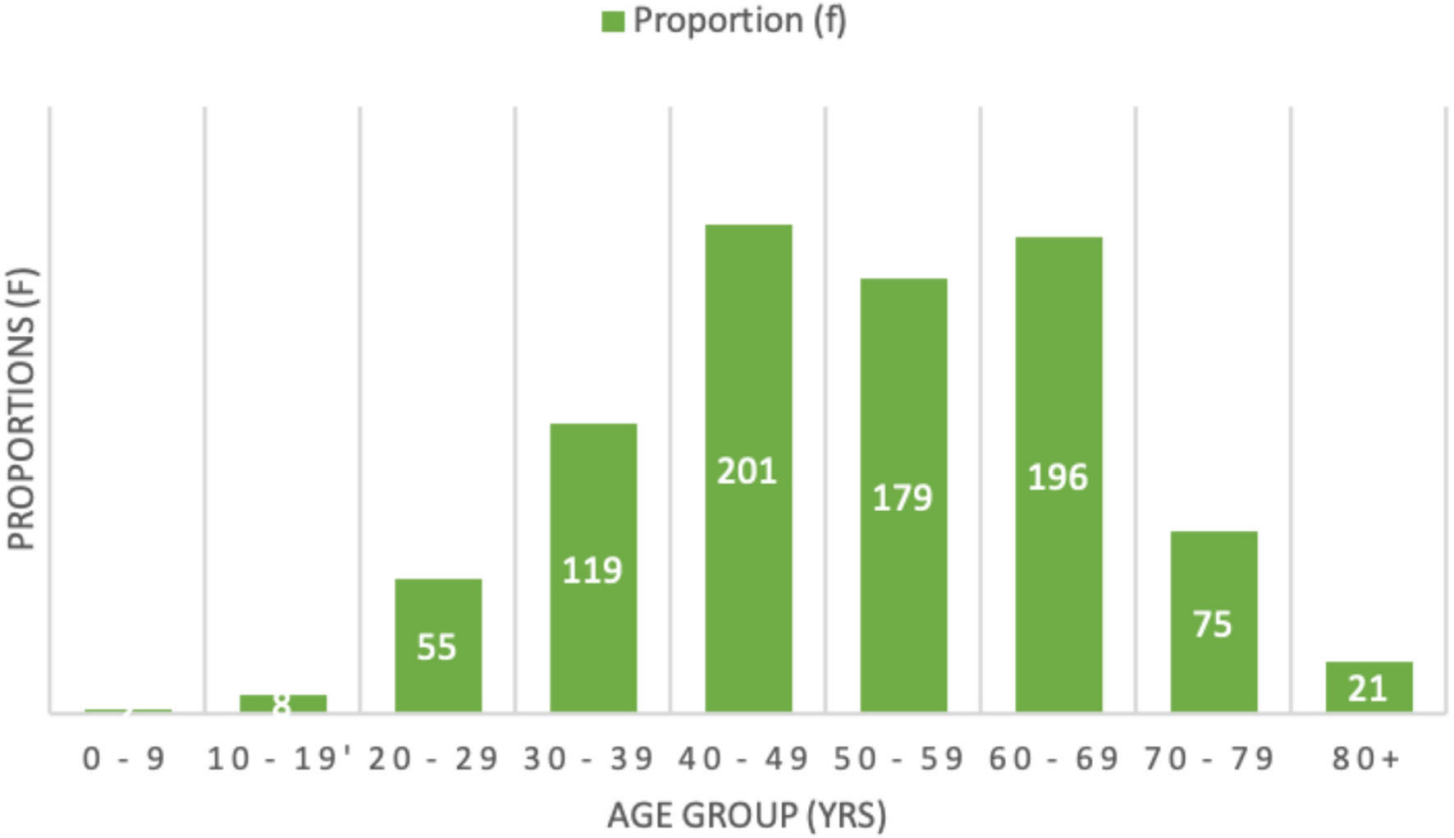
Bar charts showing the age group distribution in decades of the study population.

**Figure 2:**
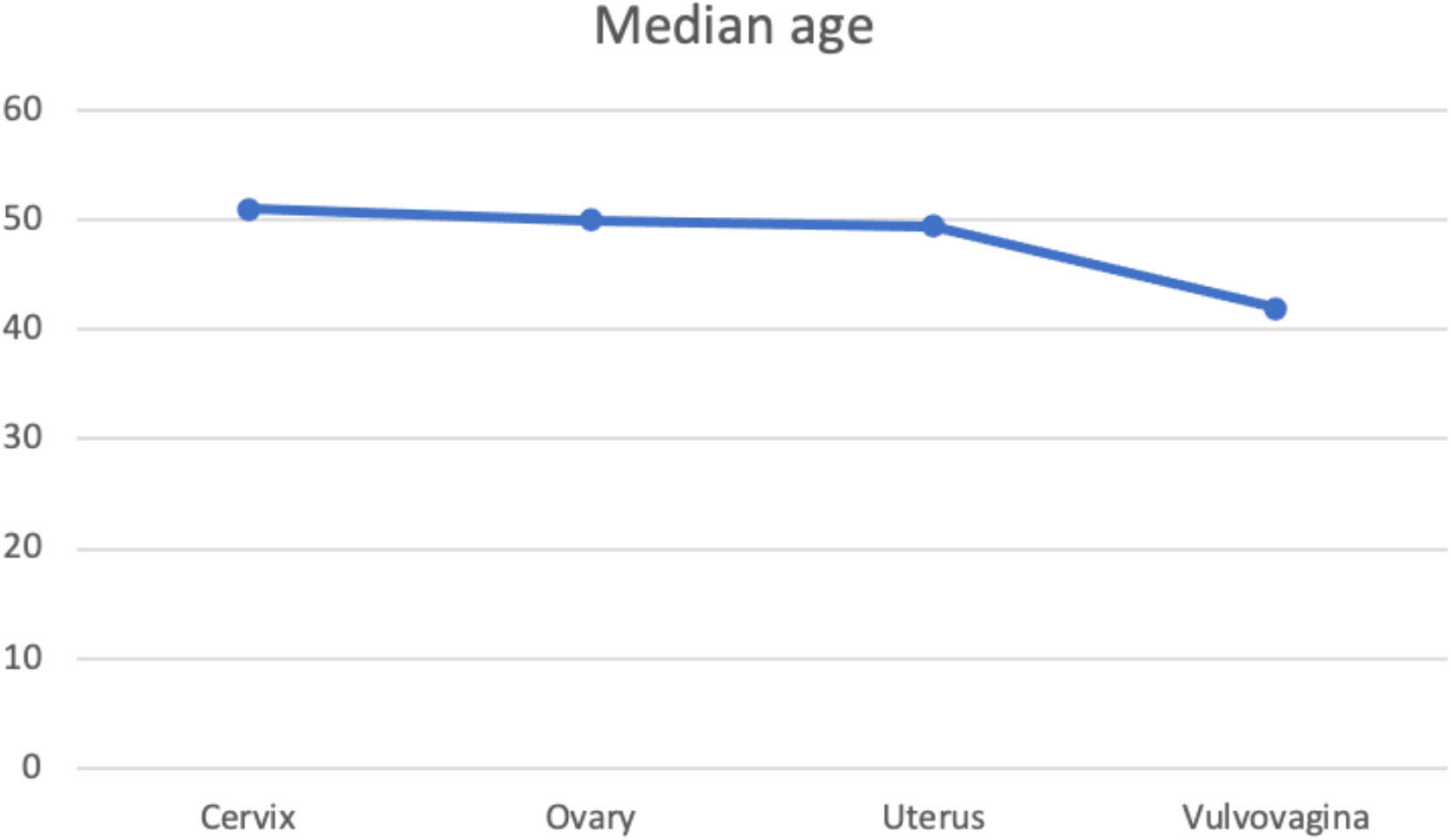
Line graph portraying the trend of median ages of patients by cancer site.

A total of 863 histologically confirmed GC were eligible for inclusion in the study. Stepwise exclusion of ineligible cases is shown in the CONSORT flowchart in **Supplementary 4**. **Figure 1** illustrates the age distribution of the patients. The median age was 50 years (range 3 – 95 years), with peak incidence in the 5^th^ decade followed by a second peak in the 7^th^ decade. **Figure 2** shows the various median ages of occurrence for the different cancer sites. Cervical cancer occurred at a higher age compared to ovarian and uterine corpus cancers, while cancers of the vagina and vulva had the least median age.

**Table 1** shows the proportions and percentages of the various cancers observed in the study period. Cervical cancer was the most common, followed by uterine cancers. A remarkable proportion of malignancies that occurred in the cervix were conveniently described as “others”, as they included not only rare tumors such as sarcomas, non-Hodgkin lymphomas, and adenoid cystic carcinoma but also because some were not outrightly classified at the time of data storage. Corpus uteri malignancies were more of endometrioid carcinoma, followed closely by choriocarcinoma. Cases of endometrial stromal sarcoma and metastatic carcinomas to the uterus were also observed. Other histological subtypes are as outlined in **Table 1**.

**Table 1:** The four broad groups of gynecologic cancers and their histological subtypes.

Organ site	Histologic subtypes	Frequency (N)	Percentage (%)
Uterine cervix	Squamous cell carcinoma	379	66
	Adenocarcinoma	58	10
	Others	138	24
Sub-Total		575	100
Uterine corpus	Choriocarcinoma	45	33.5
	Endometrioid carcinoma	48	35.8
	Squamous cell carcinoma	10	7.5
	Leiomyosarcoma	6	4.5
	Serous carcinoma	4	3.0
	Carcinosarcoma	4	3.0
	Others	17	12.7
Sub-Total		134	100
Ovary and fallopian tube	Epithelial cancers	87	68
	Sex cord stromal tumors	16	12.5
	Germ cell tumors	12	9.4
	Miscellaneous	7	5.5
	Subtype not available (NA)	6	4.6
Sub-Total		128	100
Vulvovaginal	Squamous cell carcinoma	17	65.4
	Adenocarcinoma	2	7.7
	Others	7	26.9
Sub-Total		26	100

Ovarian cancers in this study ranked third among the female genital malignancies. One case of fallopian tube cancer was seen, and this was a serous carcinoma. Together with the epithelial ovarian cancers, they constituted 67.9% (87/128) of the cancers. The various histologic variants of epithelial cancers were epithelial serous carcinoma, mucinous carcinoma, clear cell carcinoma, and endometroid carcinomas. Among the non-epithelial tumors which represented 27.3% (35/128) of all the ovarian cancers, 34.3% (12/35) were germ cell tumors (GCT), made up of immature teratoma, dysgerminoma, yolk sac tumor, choriocarcinoma and a cancer within a mature teratoma, while 45% (16/35) were of sex cord-stroma tumor in origin, all comprising of granulosa cell tumor. Few (6) cases among the ovarian cancer histotypes had no histological class designation. Thus, they could not be placed in any class. and were documented as “not available (NA)”.

Vulvovaginal cancers accounted for 26 (3.0%) cases and were the least with predominance of squamous cell carcinoma (17 cases; 65.4%).

### Gynecologic cancer presenting symptoms

The recorded history of presenting complaints was varied as there were sites with overlap in many cases. By organ sites, 87.5% and 91.7% of uterine and cervical cancer patients respectively complained of bleeding through the vagina, with coexisting foul-smelling vaginal discharge among the latter. Feeling of abdominal mass and swelling was seen among the ovarian cancer patients in about 68% of cases, 10% of these had associated vaginal bleeding. Other symptoms included weight loss, body weakness, hemoptysis, chronic pelvic pain, leakage of fecal matter, miscarriage, protrusion par vagina, vaginal nodules, bleeding vulval mass and anorexia.

## Discussion

This study has shown a remarkable burden of gynecologic malignancies that is predominantly driven by hrHPV infections (19). Our finding of more cervical cancer is similar to the study by Okunade et al. in southwest Nigeria but contrasts with a report of higher ovarian cancers in the southeast of Nigeria by Nzeribe et al. (14, 20) However, these two studies reported single tertiary institution data each, whilst the present study was multi-institutional and could represent a truer picture of the disease in the population studied. There was also a notable variation in the age of occurrence of cases among these study environments. Whereas the overall median age at diagnosis was 50 years in the present study with a peak at the 5^th^ decade, Okunade et al. in contrast, found a mean age of 52 years with a peak at the 6^th^ decade (20). These reports and that from the present study suggest that women in this population are likely to be diagnosed with gynecological cancer irrespective of subtype at a young age. Given the attendant impact of this on the economy and households, there is a need for interventions that will promote a demographic shift in the disease occurrence.

Besides low overall median age, variations were also observed among different cancer subtypes in this study. It was noted that while cervical cancer was diagnosed at a median age of 51 years, vulvovaginal cancers were diagnosed at a median age of 42 years. By histological subtype, the majority of these cancers were squamous cell carcinomas that are usually driven by a common aetiologic agent, the hrHPV (21–23). Other risk factors commonly associated with the development of these cancers, and which were likely present in this population going by the most recent census data, include high parity, early age at sexual debut, and multiple sexual partners (24–26). These cancer types also have premalignant stages that can be detected by screening and treated to forestall their transformation into a malignant disease (26). For example, vulvar intraepithelial neoplasia has two distinct subtypes, a usual type seen more in younger age groups and associated in over 80% of cases with HPV infection and a differentiated form that occurs in older women and is less HPV-dependent (27). Given the younger age of patients who had cervical, vaginal and vulval cancers in the present study, addressing issues surrounding HPV infection prevention, and early detection would help to reduce the burden of these malignancies in the region and perhaps the country at large as has been demonstrated in some Nordic countries (28). A population-specific approach may be needed in the African population as data suggests differences in hrHPV prevalence among the black population compared to White women (29). Also, the absence of the cervix, as in women who had undergone a hysterectomy, should not preclude vaccination against hrHPV as studies have shown that they can develop lower genital tract HPV-associated cancers, such as vaginal, vulval and anal cancers in the future (30).

The finding of more epithelial cancer among ovarian cancers and at a young age in this study is in keeping with the literature (18). We suspect from the census data that the protective influence of high parity against ovarian cancers among women in this region could account for the overall low proportion of this group of malignant diseases in this study (31). Non-epithelial tumors, on the other hand, though still low in proportion, exceeded data documented in other studies (32). Whereas this may not represent an increase in incidence of cases, nevertheless, the age at diagnosis is in keeping with the age demographics reported by other studies (32).

Uterine cancer had similar incidence as ovarian cancer in this study, although it surpasses cervical and ovarian cancers in developed countries (19). Emerging evidence suggests that African American or Black women have both higher incidence and worse mortality from the disease than White women in the United States, suggesting racial disparity in disease occurrence that may be explained by differing genetic predispositions (33, 34). For example, Black women have been shown to have a much greater risk of high-grade (serous and carcinosarcoma) uterine cancers relative to White women. In the same cohort, copy-number high (serous-like) tumors had a direct correlation with percent African ancestry (35). Efforts to reduce the more readily modifiable risk factors of worse outcomes among Black women, such as low health education (including awareness of symptoms), late detection, treatment delays, and poor implementation of evidence-based treatment recommendations, should be prioritized to improve the women’s health in this regard (36).

The high proportion of cases of gestational choriocarcinoma in this study raises significant concerns regarding the needs of the local population who develop these aggressive yet chemotherapy-sensitive pregnancy-related cancers, which could threaten their lives during their reproductive years. An explanation for this finding in this study is unclear. However, a review of literature on gestational choriocarcinoma across different regions globally showed that older maternal age, long-term oral contraceptive use and socioeconomic status were strong risk factors for this cancer subtype (37). While these risk factors have not been investigated presently, we recommend a high index of alertness by gynecologists to be able to salvage all such patients. Campaigns to encourage ante- and postnatal care services utilization are also expedient so that more women can be brought into the “safe net” of gynecologic care within which such adverse pregnancy outcomes can be detected and treated early.

The absence of data on tumor staging, treatment received and survival limits further analysis of the impact of the disease on the population. Recent studies from northern Nigeria revealed that about 67%-92% of women with GC present at a very advanced stage (38, 39). The region also suffers from a lack of skilled specialists for oncology care. Northeastern Nigeria has the lowest number of obstetricians and gynecologists in the country (40). This has implications on whether the patients are diagnosed at all or on time. Indeed, it has been shown that about 80% of patients experience a delay in diagnosis within a hospital setting, which could be due to prolonged waiting time on account of high physician workload (38). Another area of concern is the availability of care and its accessibility. The predominant form of treatment fo GC in this region are surgery and chemotherapy, which the majority are not able to afford out-of-pocket (38, 39, 41). Radiation oncology services are rarer and have a very long waiting time (42). These care gaps warrant policy guidelines to address. Training of more manpower is urgently needed to detect cases early while strengthening GC care through infrastructural provision and universal health insurance coverage.

Other limitations to this study include the non-population level data reporting and incomplete classification of some cases according to sites of origin. Likewise, the non-inclusion of clinically diagnosed cases and non-documentation of the Human Immunodeficiency Virus infection status of the patients with cervical, vaginal and vulvar cancers, all affect accurate estimation of the burden and risk factors of the disease in the population. Future studies should be designed in such a way to capture these and determine the financial, social and psychological impact of the disease on the women and their families. This will help to determine how to support them along their care journey (43).

## Conclusion

The spectrum of gynecologic cancers in this study reveals a need for HPV infection prevention through advocacy, vaccination, screening and treatment of premalignant lesions. While these will reduce the cases of cervical, vulva and vaginal cancers, health education to the women regarding possible symptoms and signs of endometrial and ovarian cancers will enable them to present early to hospital for care. Future research is required to determine the risk factors for endometrial and ovarian cancers in this population, alongside investigating the impact of GC on the women. Lastly, population-specific policy direction that takes into account required manpower and infrastructural need to curb GC morbidity and mortality is highly advocated.

## Data Availability

The original contributions presented in the study are included in the article/**Supplementary Material**. Further inquiries can be directed to the corresponding author.
