# Supplementary material for: Spectrum of gynecologic malignancies in Northeastern Nigeria
Source: Front Oncol. 2025 Mar 18;15:1420113. doi: 10.3389/fonc.2025.1420113 (PMC11959032; doi:10.3389/fonc.2025.1420113)
Supplement: Supplementary file 1 [file DataSheet1.zip › Supplementary 1.DOCX]

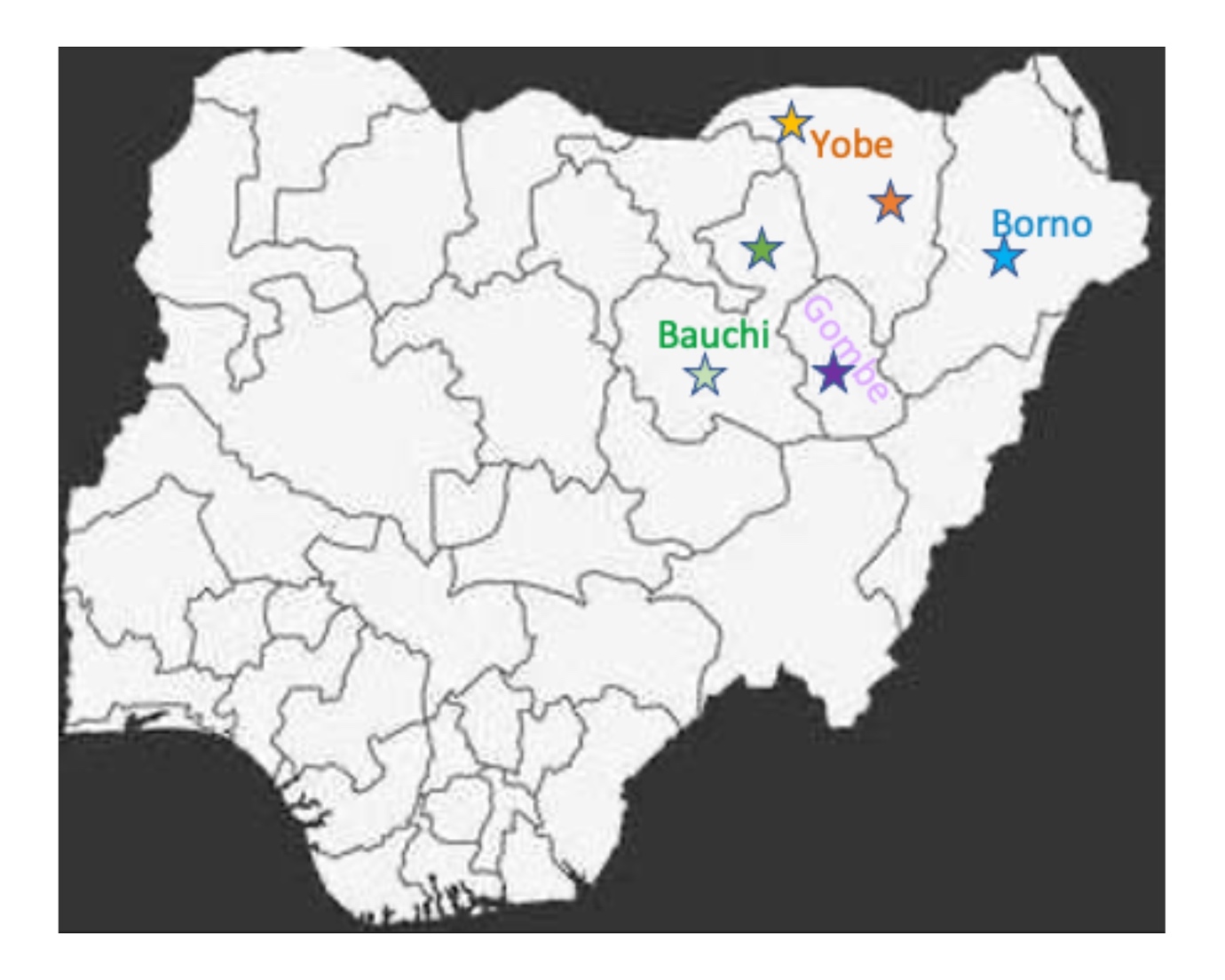


**Supplementary 1: Map of Nigeria showing the four (4) northeast states of Borno, Yobe, Bauchi and Gombe and location of the participating hospitals. Federal Medical Centre (FMC) Nguru; Yobe State University Teaching Hospital (YSUTH); University of Maiduguri Teaching Hospital (UMTH); Federal Medical Centre (FMC) Azare; Abubakar Tafawa Balewa University Teaching Hospital (ATBUTH); Federal Teaching Hospital, (FTH) Gombe.**

D

D

D

D

D

D
