# Supplementary material for: Spectrum of gynecologic malignancies in Northeastern Nigeria
Source: Front Oncol. 2025 Mar 18;15:1420113. doi: 10.3389/fonc.2025.1420113 (PMC11959032; doi:10.3389/fonc.2025.1420113)
Supplement: Supplementary file 1 [file DataSheet1.zip › Supplementary 2.DOCX]

Supplementary 2: Population distribution of females in the study area and their age profile categorized by fertility period 0-49 years and above**.**

| **States** | **Total** | **Females** | **0 – 49 years** | **50 – 80+ years** |
| --- | --- | --- | --- | --- |
| **Bauchi** | 8,308,783 | 4,160,248 | 3,864,571 | 295,677 |
| **Borno** | 6,111,462 | 3,029,395 | 2,757,846 | 271,549 |
| **Gombe** | 3,960,122 | 1,907,881 | 1,761,745 | 146,136 |
| **Yobe** | 3,649,607 | 1,794,832 | 1,654,329 | 140,503 |
| **Total** | **22,029,974** | **10892356** | **10,038,491** | **853,865** |
