# Supplementary material for: Spectrum of gynecologic malignancies in Northeastern Nigeria
Source: Front Oncol. 2025 Mar 18;15:1420113. doi: 10.3389/fonc.2025.1420113 (PMC11959032; doi:10.3389/fonc.2025.1420113)
Supplement: Supplementary file 1 [file DataSheet1.zip › Supplementary 4.DOCX]

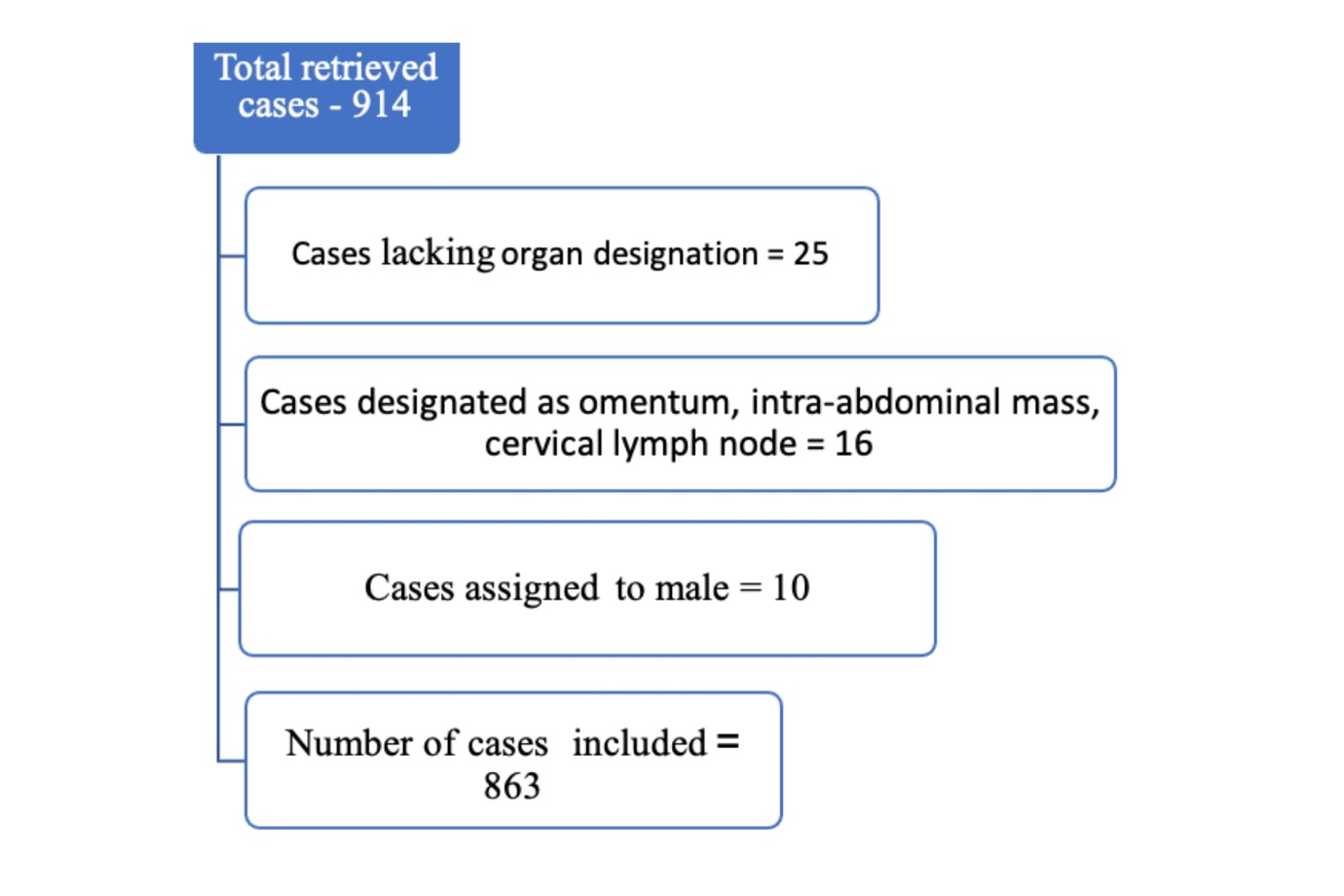


Supplementary 4: CONSORT flow chart showing stepwise exclusion of ineligible cases from the obtained data
