# Supplementary figures and images for: Spectrum of gynecologic malignancies in Northeastern Nigeria
Source: Front Oncol. 2025 Mar 18;15:1420113. doi: 10.3389/fonc.2025.1420113 (PMC11959032; doi:10.3389/fonc.2025.1420113)

**
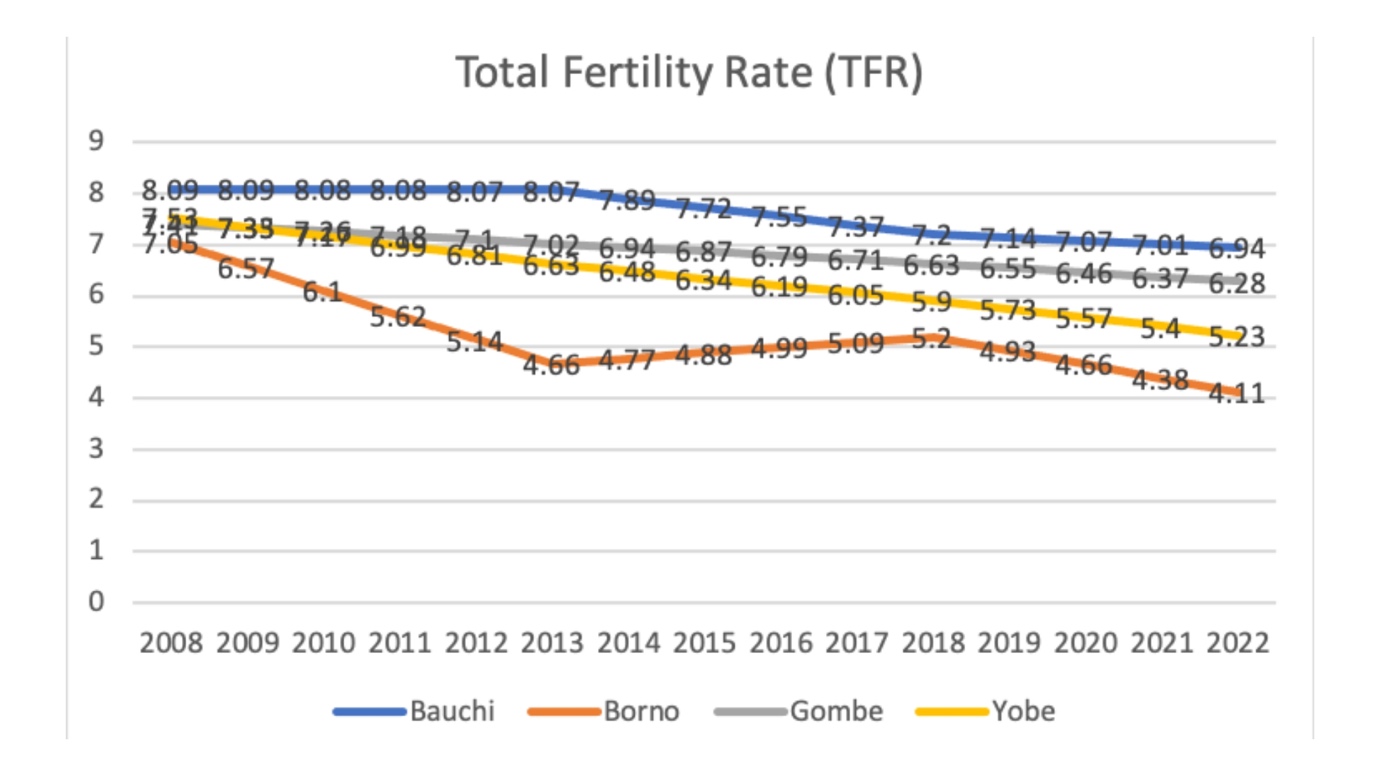
**

**Supplementary 3: Trend in Total Fertility Rate (TFR) among females in the zone.**

Supplement: Supplementary file 1 [file DataSheet1.zip › Supplementary 3.DOCX]
